# miR-124-3p and miR-194-5p regulation of the PI3K/AKT pathway via ROR2 in medulloblastoma progression

**DOI:** 10.1038/s41417-024-00762-y

**Published:** 2024-03-19

**Authors:** Chen Wang, Runxi Fu, Yunkun Wang, Jia Wei, Ying Yu, Liuhua Hu, Chenran Zhang

**Affiliations:** 1 Department of Pediatric Neurosurgery, Xinhua Hospital, Shanghai Jiao Tong University School of Medicine, Shanghai, China; 2 Department of Pediatric Surgery, Xinhua Hospital, Shanghai Jiao Tong University School of Medicine, Shanghai, China; 3 Shanghai Institute for Pediatric Research, Shanghai, China; 4 Department of Cardiology, Renji Hospital, Shanghai Jiao Tong University School of Medicine, Shanghai, China

**Keywords:** CNS cancer, Paediatric cancer

## Abstract

Medulloblastoma (MB), a prevalent pediatric central nervous system tumor, is influenced by microRNAs (miRNAs) that impact tumor initiation and progression. However, the specific involvement of miRNAs in MB tumorigenesis remains unclear. Using single-cell RNA sequencing, we identified ROR2 expression in normal human fetal cerebellum. Subsequent analyses, including immunofluorescence, quantitative real-time PCR (qRT-PCR), and Western blot, assessed ROR2 expression in MB tissues and cell lines. We investigated miR-124-3p and miR-194-5p and their regulatory role in ROR2 expression through the dual-luciferase reporter, qRT-PCR, and western blot assays. Mechanistic insights were gained through functional assays exploring the impact of miR-124-3p, miR-194-5p, and ROR2 on MB growth in vitro and in vivo. We observed significantly reduced miR-124-3p and miR-194-5p expression and elevated ROR2 expression in MB tissues and cell lines. High ROR2 expression inversely correlated with overall survival in WNT and SHH subgroups of MB patients. Functionally, overexpressing miR-124-3p and miR-194-5p and inhibiting ROR2 suppressed in vitro malignant transformation and in vivo tumorigenicity. Mechanistically, miR-124-3p and miR-194-5p synergistically regulated the ROR2/PI3K/Akt pathway, influencing MB progression. Our findings indicate that miR-124-3p and miR-194-5p function as tumor suppressors, inhibiting MB progression via the ROR2/PI3K/Akt axis, suggesting a key mechanism and therapeutic targets for MB patients.

## Introduction

Medulloblastoma (MB) is the most common malignant neoplasm of the brain in childhood, constituting 15–30% of neurological tumors [1, 2]. Advances in multi-modality therapies over the past 20 years have improved the overall 5-year survival rate of children with MB from 60 to 70% [3, 4]. However, the long-term sequelae caused by surgery, chemotherapy, and radiotherapy have led to increased incidences of tumor recurrence, neurocognitive dysfunction, endocrine deficiencies, and incapacitating chronic diseases in survivors [5, 6].

With the evolution of cancer genomics, MB classifications have been refined, and the World Health Organization classifications have been periodically updated based on molecular and histological characteristics [7–9]. Despite inconsistencies in the precise origin/structure of molecular MB subgroups, four core subgroups [WNT, SHH, Group 3 (G3), and Group 4 (G4)] are internationally recognized [10–12]. Most WNT subtypes exhibit somatic point mutations in the CTNNB1 gene, encoding the β-catenin protein. The SHH subtype frequently displays activating mutations in the PTCH1, SMO, and SUFU genes. However, specific biomarkers for Group 3 and Group 4 MB necessitate further investigation. New effective subgroup-specific therapies targeting distinct molecular markers are being developed to enhance the outlook for MB-affected children [13]. However, each MB subgroup presents unique heterogeneous transcription profiles, rendering treatments diverse and complicating drug development [1]. Thus, the identification of a common drug targeting homogeneously expressed biomarkers across the four MB subgroups holds pivotal significance in enhancing survival rates and mitigating postoperative toxicity for survivors [10, 14].

Receptor tyrosine kinase-like orphan receptor 2 (ROR2) is a member of the receptor tyrosine kinase superfamily, acting as a developmentally regulated kinase in humans [15, 16]. Throughout ontogeny, ROR2 displays dynamic regulation from embryo to adult. During early organogenesis, ROR2 exhibits significant expression in various tissues, including skeletal, brain, ocular epithelium, respiratory, and cardiac systems [17–19]. However, during midgestation and the neonatal period, ROR2 is significantly downregulated in multiple organs. In adulthood, ROR2 is nearly undetectable in most tissues, except specific compartments in the colon, osteoblasts, and thyroid [20–22]. Interestingly, ROR2 is re-expressed in a wide range of adult cancers, such as prostate carcinoma, oral squamous cell carcinoma, melanoma, colorectal cancer, and osteosarcoma, playing significant roles in tumor cell metabolism, proliferation, invasion, and metastasis [23–25]. Given these crucial attributes and its emerging association with oncogenesis, ROR2 has garnered attention as a central target for therapeutic intervention [26–28]. However, the precise mechanisms by which ROR2 functions in the tumorigenesis of pediatric MB remain incompletely understood.

The phosphatidylinositol 3-kinase (PI3K)/Akt pathway is a vital intracellular signaling cascade governing multiple downstream biological functions [29, 30]. This pathway is increasingly recognized as contributing to a spectrum of cancers, including gastric cancer, colon cancer, osteosarcoma, and glioblastoma [31–34]. Dysregulation of the PI3K/Akt pathway in tumor cells influences various tumorigenesis processes, encompassing proliferation, apoptosis, angiogenesis, metastasis, and drug resistance [35–37]. Highly activated PI3K/Akt signaling has been linked to MB progression. Conversely, suppressing Akt phosphorylation reduces MB proliferation, migration, and radiation resistance [38–40]. In the context of the regulatory relationship between ROR2 and Akt, studies have indicated that ROR2 can modulate Akt phosphorylation through activation or inhibition in different cancers, including melanoma, breast cancer, myeloma, and osteosarcoma [41–44]. However, the precise role of ROR2 in MB tumorigenesis via Akt regulation remains elusive.

MicroRNAs (miRNAs) are endogenous small non-coding RNAs that modulate gene expression, consisting of approximately 19–21 nucleotides [44]. Mature miRNAs assemble into the RNA-induced silencing complex (RISC), complementarily binding to the 3′-untranslated region (3’UTR) of target mRNAs for degradation [45, 46]. Disrupted miRNA expression induces alterations in gene profiles, contributing to diverse disease pathogenesis [47, 48]. miRNAs are promising biomarkers for disease diagnosis, prognosis, and treatment due to their robust stability in human circulation [49, 50]. miR-124-3p and miR-194-5p, recognized as neural-related miRNAs, have roles in central nervous system cancers such as astrocytoma and glioblastoma [51–53]. However, their expression and functions in MB remain unclear.

This study presents the downregulation of miR-124-3p and miR-194-5p in pediatric MB and their role as tumor suppressors through their synergistic upregulation of ROR2. Elevated ROR2 inhibited apoptosis and enhanced cell proliferation, migration, and invasion to drive MB growth through the PI3K/AKT pathway. Our findings provide insights into the oncogenesis, prognosis prediction, and therapeutic targeting of MB.

## Methods

### Human medulloblastoma tissue samples

In this study, samples of 14 cases with primary pediatric medulloblastoma and 9 normal control cases, which consisted of 8 subjects of adjacent normal tissues and 1 subject of epilepsy, were obtained from patients who underwent surgery in the Xinhua Hospital of Shanghai Jiao Tong University. All MB cases were diagnosed by histopathological examination of biopsy/surgical resection material. The clinical samples were quickly frozen in liquid nitrogen after operation and then stored at −80 °C until use. The present study was conducted in accordance with the Helsinki Declaration and approved by the Xinhua Hospital Ethics Committee (XHEC-D-2021-087), and all patients were provided with the consent forms signed by their parents. The detailed clinical information of patients is summarized in Table 1.

**Table 1:** Clinical features of medulloblastoma patients.

No.	Sex	Age (m)	Histology	Subgroup
1	M	15	Desmoplastic/nodular	SHH
2	F	13	Classic	Group 4
3	F	103	Classic	WNT
4	F	124	Classic	WNT
5	M	72	Classic	Group 3
6	F	77	Classic	SHH
7	F	60	Desmoplastic/nodular	Group 4
8	M	57	Classic	WNT
9	M	35	Classic	Group 4
10	F	30	Desmoplastic/nodular	Group 3
11	F	39	Classic	SHH
12	F	24	Classic	Group 4
13	M	102	Classic	Group 4
14	M	157	Classic	Group 4

### Single-cell RNA sequencing datasets and analysis

For human fetal cerebellums, the DESCARTES platform was used to analyze single-cell atlases of gene expression, which is based on a three-level single-cell combinatorial indexing assay for gene expression (sci-RNA-seq3) to 121 human fetal samples [54].

### Immunofluorescence staining (IF)

MB and control tissue were fixed in 4% paraformaldehyde, permeabilized with 0.5% Triton X-100, embedded in paraffin, and sectioned at 5 μm. After incubation with primary antibody specific for ROR2 (1:200, Cell Signaling Technology, USA) at 4 °C overnight, the sections were then incubated with anti-rabbit secondary antibodies (Cell Signaling Technology, USA) for 1 h at room temperature. Nuclei were stained with the DAPI (Cell Signaling Technology, USA). The samples were visualized with a fluorescence microscope.

### RNA extraction and real-time quantitative real-time polymerase reaction (qRT-PCR)

Total RNA was extracted from tissues or cells using TRIzol Reagent (Takara, Japan) according to the manufacturer’s instructions. The concentration and purity of RNA samples were measured by Nanodrop 2000 instrument (Thermo Fisher Scientific, USA). For mRNA analysis, the reverse transcription reactions were performed using the High Capacity cDNA Reverse Transcription Kit (Thermo Fisher Scientific, USA), and qRT-PCR was performed using SYBR Green Real-Time PCR Master Mixes (Thermo Fisher Scientific, USA). GAPDH was applied as an endogenous standard control for normalization. For miRNA analysis, the complementary deoxyribonucleic (cDNA) was obtained using the TaqMan MicroRNA Reverse Transcription Kit (Thermo Fisher Scientific, USA), and qRT-PCR was performed using TaqMan Universal Master Mix II (Thermo Fisher Scientific, USA). U6 small nuclear RNA (U6 snRNA) was applied as an endogenous standard control for normalization. The relative quantification of RNA expression levels was analyzed using the 2^-ΔΔCT^ method. The primers are listed in Table 2.

**Table 2:** Primer sequences used in this study.

Gene name	Primer type	Sequence (5′ − 3′)
ROR2	Forward	CCTGGTGCTTTACGCAGAATA
	Reverse	TGGGGACCAAGATGTACAGAA
GAPDH	Forward	GAGTCAACGGATTTGGTCGT
	Reverse	TGTGGTCATGAGTCCTTCCA
miR-124-3p	Forward	TAAGGCACGCGGTGAATGCCAA
	Reverse	Provided by manufacturer
miR-194-5p	Forward	TGTAACAGCAACTCCATGTGGA
	Reverse	Provided by manufacturer
U6	Forward	GCTTCGGCAGCACATATACTAAAAT
	Reverse	CGCTTCACGAATTTGCGTGTCAT

### Western blotting analysis (WB)

Tissue and cells were lysed in RIPA Lysis Buffer (Thermo Fisher Scientific, USA) on ice. The total protein concentration was measured and quantified by BCA protein assay (Beyotime, China). The equal amounts of total protein from different samples were separated by SDS-PAGE gels and transferred onto polyvinylidene fluoride (PVDF) membranes (Millipore, USA). The membranes were blocked with 5% nonfat milk and incubated with specific primary antibody anti-ROR2 (1:1000, Cell Signaling Technology, USA), anti-AKT (1:1000, Cell Signaling Technology, USA), anti-p-AKT (Ser473) (1:1000, Cell Signaling Technology, USA), anti-Caspase-3 (1:2500, Cell Signaling Technology, USA), anti-Cleaved Caspase-3 (1:2000, Cell Signaling Technology, USA), anti-PARP (1:2500, Cell Signaling Technology, USA), anti-Cleaved PARP (1:1500, Cell Signaling Technology, USA), anti-Caspase-9 (1:3000, Cell Signaling Technology, USA), anti-Cleaved Caspase-9 (1:1500, Cell Signaling Technology, USA), anti-Bad (1:2000, Abcam, USA), anti-Bax (1:2000, Abcam, USA), anti-Bcl-2 (1:2500, Abcam, USA), anti-Bcl-XL (1:3000, Abcam, USA), anti-MCL1 (1:3000, Abcam, USA), anti-Vimentin (1:2000, Cell Signaling Technology, USA), anti-N-Cadherin (1:2500, Cell Signaling Technology, USA), anti-Claudin-1 (1:1500, Cell Signaling Technology, USA), anti-Snail (1:2000, Cell Signaling Technology, USA), anti-Slug (1:2000, Cell Signaling Technology, USA), anti-E-Cadherin (1:2500, Cell Signaling Technology, USA) and anti-GAPDH (1:5000, Cell Signaling Technology, USA) at 4 °C overnight. Then, the membranes were washed with 1×TBST and incubated with anti-rabbit or anti-mouse HRP-conjugated secondary antibodies (1:5000, Cell Signaling Technology, USA) for 1.5 h at room temperature. The membranes were visualized by an enhanced chemiluminescence kit (Thermo Fisher Scientific, USA). GAPDH was used as an endogenous standard control for normalization. The images were analyzed by Image Lab Software.

### Cell culture and transfection

The human medulloblastoma cell lines DAOY, D283 Med, and D341 Med were purchased from the American Type Culture Collection (ATCC, USA). Untransformed Normal Human Astrocytes (NHA) were purchased from CMBIO (Shanghai, China). DAOY and D283 Med cell lines were cultured in Eagle’s Minimum Essential Medium (EMEM, Corning, USA) with 10% FBS (Gibco, USA), 1% penicillin (100 U/ml) and 0.1 mg/ml streptomycin (Gibco, USA). D341 Med cell line was cultured in Minimum Essential Medium with Earle’s Balanced Salt Solution (MEM with EBSS, Cytiva, USA) with 10% FBS (Gibco, USA), 1% penicillin (100 U/ml) 0.1 mg/ml streptomycin (Gibco, USA), 1% 100× NEAA (Thermo Fisher Scientific, USA) and 1 mM sodium pyruvate (Thermo Fisher Scientific, USA). NHA was cultured in Dulbecco’s Modified Eagle’s medium (DMEM, Corning, USA) with 10% FBS (Gibco, USA), 1% penicillin (100 U/ml), and 0.1 mg/ml streptomycin (Gibco, USA). All cells were grown in a humidified chamber with 5% CO_2_ and 95% air at 37 °C. The miR-124-3p mimics, miR-194-5p mimics, mimics nc, miR-124-3p inhibitor, miR-194-5p inhibitor, inhibitor nc, siROR2 and control siRNA were designed and synthesized by GenePharma (Shanghai, China). The miR-124-3p agomir, miR-194-5p agomir, ROR2-shRNA, and controls were designed and synthesized by Genomeditech (Shanghai, China). Lipofectamine 2000 reagent (Thermo Fisher Scientific, USA) was used as transfection medium according to the manufacturer’s guideline. Oligonucleotide sequences are listed in Table 3.

**Table 3:** Sequence of oligonucleotide used in this study.

Gene name	Primer type	Sequence (5′ − 3′)
siROR2-1	Sense	GGAUCAUCAUCCGGAAGACTT
	antisense	GUCUUCCGGAUGAUGAUCCTT
siROR2-2	Sense	CCGCUACCAUCAGUGCUAUTT
	antisense	AUAGCACUGAUGGUAGCGGTT
siROR2-3	Sense	GACAGAAUAUGGUUCACGATT
	antisense	UCGUGAACCAUAUUCUGUCTT
NC	Sense	Provided by the manufacturer
	antisense	
miR-124-3p mimics	Sense	UAAGGCACGCGGUGAAUGCC
	antisense	CAUUCACCGCGUGCCUUAUU
miR-124-3p inhibitor	Sense	GGCAUUCACCGCGUGCCUUA
	antisense	/
miR-194-5p mimics	Sense	UGUAACAGCAACUCCAUGUGGA
	antisense	CACAUGGAGUUGCUGUUACAUU
miR-194-5p inhibitor	Sense	UCCACAUGGAGUUGCUGUUACA
	antisense	/
mimics NC	Sense	Provided by the manufacturer
	antisense	
inhibitor NC	Sense	Provided by the manufacturer
	antisense	

### Dual-luciferase reporter assay

The Luciferase reporter vector of ROR2–3’UTR and its corresponding mutation were constructed by Genechem (Shanghai, China), including ROR2–3’UTR-WT and ROR2–3’UTR-MUT. DAOY cells were seeded in 24-well plates in triplicate and co-transfected with corresponding plasmids and miR-124-3p mimics, miR-194-5p mimics or mimics NC. Then, after 48 h of incubation, the Firefly and Renilla luciferase activities were measured by Dual Luciferase Assay Kit (Promega, USA) according to the manufacturer’s protocols. Relative luciferase activity was normalized to the Renilla luciferase internal control.

### Wound-healing assay

Cells were seeded in six-well plates and incubated with drugs for an additional 24 h until they reached about 90–95% confluence. Afterward, a vertical scratch wound was created in the cell monolayer of each well’s central area using a 200-μl pipette tip. Subsequently, cells were washed with 1× PBS twice and then cultured in the serum-free MEM medium. The wound closure photographs were taken at 0 h and 24 h after injury (objective lens: ×10; eyepiece: ×10). Cell migration was quantified following the equation: (0 h wound area−24 h wound area)/0 h wound area × 100. The images were analyzed by ImageJ Software. Each experiment was performed in triplicate.

### Transwell migration and invasion assays

For measure the abilities of cells’ invasion and migration after transfection, transwell chambers (8-μm pore size, Corning, USA) were paved with Matrigel (Corning, USA) or without. After transfection with drugs for 48 h, the cells were washed by 1× PBS twice and resuspended in their corresponding serum-free culture medium (1 × 10^6^ cells/mL). Then, 800 μl medium with 10% FBS was added to the bottom chamber, and 100 μl of the suspension was seeded into the upper chamber. After incubation for 24 h at 37 °C, the upper chambers were fixed with a 4% paraformaldehyde for 30 min and stained with 1% crystal violet solution for 15 min. The images were analyzed by ImageJ Software. Three visual fields were randomly selected for manual counting (objective lens: ×10; eyepiece: ×10). Each experiment was performed in triplicate.

### Colony formation assay

Cells were seeded into 6-well plates at a density of 500 cells per well and cultured with 5% CO_2_ and 95% air at 37 °C for 2 weeks. The medium was refreshed every three days. Then, the colonies were fixed with a 4% paraformaldehyde for 30 min and stained with 1% crystal violet solution for 15 min, and pictures were taken and counted. The colonies' photographs were taken and counted. Each experiment was performed in triplicate.

### Cell counting kit-8 proliferation assay

Cell proliferative capacity in different groups was measured by CCK-8 reagent (Yeasen, China). A total of 3000 cells were seeded in each well of a 96-well plate. Before the detection of the optional density at 450 nm by an automatic microplate reader (Bio-Rad, USA), each wells were added with 10 μL of CCK-8 reagent and incubation at 37 °C for 1 h. Cell proliferation was recorded at different time points (24, 48, and 72 h). Each experiment was performed in triplicate.

### Cell apoptosis assay

Cell apoptosis was detected by Annexin V-FITC/propidium iodide (PI) kit (BD Biosciences USA) and measured by flow cytometer (FACSCalibur, USA). After transfection with drugs for 24 h, the cells were digested by trypsin, washed by ice-cold 1× PBS and stained with 5 μl FITC and 5 μl PI in the dark for 15 min. The results were analyzed by FlowJo software (Tree Star, USA).

### Mouse xenograft model

Six-week-old female nude mice were chosen for establishing the xenograft model. DAOY cells were stably transfected with shROR2, miR-124-3p agomir, miR-194-5p agomir, and shNC, respectively. Then, each nude mouse was subcutaneously injected with 1 × 10^7^ DAOY cells in the flank region (10 mice per group). Tumor volumes were measured every 7 days and calculated using the following formula: volume =1/2 (length × width^2^). After 28 days, all the mice were sacrificed, and then the xenograft tumors were resected and collected. All animal care and experiments were performed according to the guidelines of the National Institutes of Health and approved by the Animal Care and Use Committees of Xinhua Hospital of Shanghai Jiao Tong University School of Medicine (XHEC-F-2023-037).

### Statistical analysis

Statistical analyses were performed using the GraphPad Prism 8.02 (GraphPad Software, USA). All data were presented as mean ± SEM. Two-tailed Student’s *t* tests were used to calculate statistical significance. The Pearson correlation coefficient was used to analyze the correlations. *P* value < 0.05 was considered statistically significant.

## Results

### Elevated ROR2 expression in MB correlates with an unfavorable prognosis

Although the cell-of-origin for MB remains incompletely elucidated, prior research has suggested that the WNT, SHH, and G4 subgroups may originate from radial glia, granule neuron progenitors, and unipolar brush cell populations [55, 56]. Furthermore, during neurological development from the embryonic to neonatal stages, early and late radial glial cells differentiate into astrocytes and oligodendrocytes [57, 58].

**Fig. 1:**
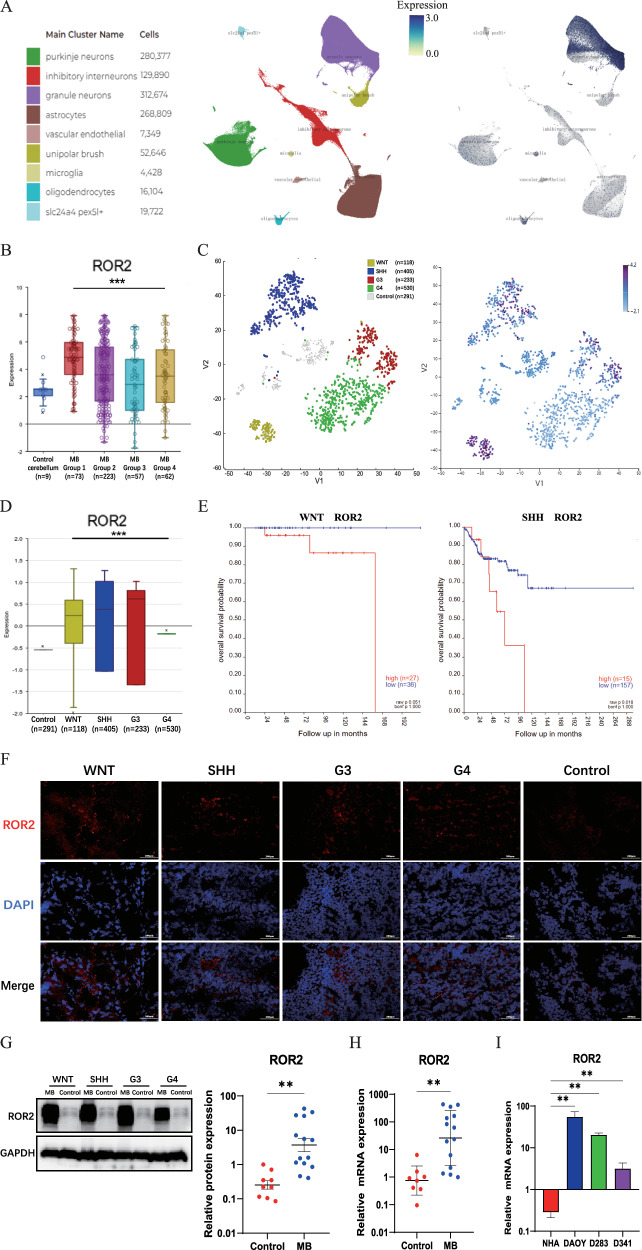
Elevated ROR2 expression in MB correlates with an unfavorable prognosis. **A** Uniform Manifold Approximation and Projection (UMAP) visualization and marker-based annotation of cells from the human fetal cerebellum colored by cell type (left panel). Plots in the right panel were colored by the normalized expression of cell-type-specific ROR2 in the human fetal cerebellum. **B** Expression analysis for ROR2 in MB and control groups using the R2 platform. Control cerebellum, Roth et al., 2008 (*n* = 9, GSE3526); MB Group1, Pfister et al., 2015 (*n* = 73, GSE49243); MB Group2, Pfister et al., 2017 (*n* = 223); MB Group 3, Delattre et al., 2012 (*n* = 57); MB Group 4, Kool et al., 2009 (*n* = 62, GSE10327). **C**, **D** Expression analysis for ROR2 in WNT, SHH, G3, and G4 groups using the R2 platform. Swartling et al., 2021 (*n* = 1641, GSE124814). **E** Survival analysis for ROR2 in WNT and SHH subgroup using the R2 platform. Cavalli et al., 2017 (*n* = 763, GSE85217). **F** IF analysis of ROR2 in MB subgroups and control tissues. **G** Western blot analysis of ROR2 protein in MB and control tissues. **H** qRT-PCR analysis of relative ROR2 mRNA expression from MB and control tissues. **I** qRT-PCR analysis of relative ROR2 mRNA expression in MB cell lines and NHA (normal human astrocytes). Data are shown as mean ± SEM; *P < 0.05, **P < 0.01, ***P < 0.001.

To explore this further, we initially utilized an online genomics analysis and visualization platform (Descartes) to delineate the distinct expression profiles of ROR2 during human cerebellum development. In the single-cell atlases derived from human fetal cerebella obtained during midgestation [54], ROR2 exhibited robust expression in astrocytes, oligodendrocytes, granule neurons, and unipolar brush cells (Fig. 1A). Furthermore, ROR2 displayed pronounced overexpression in MB across diverse ethnic groups, as demonstrated by data obtained from the online genomics analysis and visualization platform (R2) (Fig. 1B).

Subsequently, we assessed ROR2 expression in the four MB subgroups. Notably, high expression of ROR2 was observed across the SHH, WNT, G3, and G4 groups (Fig. 1C, D). Elevated ROR2 expression negatively impacted overall survival specifically in the WNT and SHH subgroups (Fig. 1E), even though the raw *P* value within the WNT group was only marginally significant at 0.051.

Next, we undertook a comparative analysis of ROR2 expression between our MB and control cerebellar samples. Comprehensive evaluation through Immunofluorescence, Western blot, and qRT-PCR assays consistently revealed significant upregulation of ROR2 in all four subgroups of MB tissues compared to normal cerebellar tissue (Fig. 1F, G). In addition, we confirmed heightened ROR2 expression in specific MB cell lines: SHH-type (DAOY), G3/4-type (D283), and G3-type (D341) (Fig. 1I). Collectively, our data demonstrate the prominent upregulation of ROR2 across the four MB subgroups, suggesting its potential role in MB tumorigenesis.

### Inhibition of ROR2 suppresses proliferation and induces apoptosis via the PI3K/AKT pathway in MB cells

**Fig. 2:**
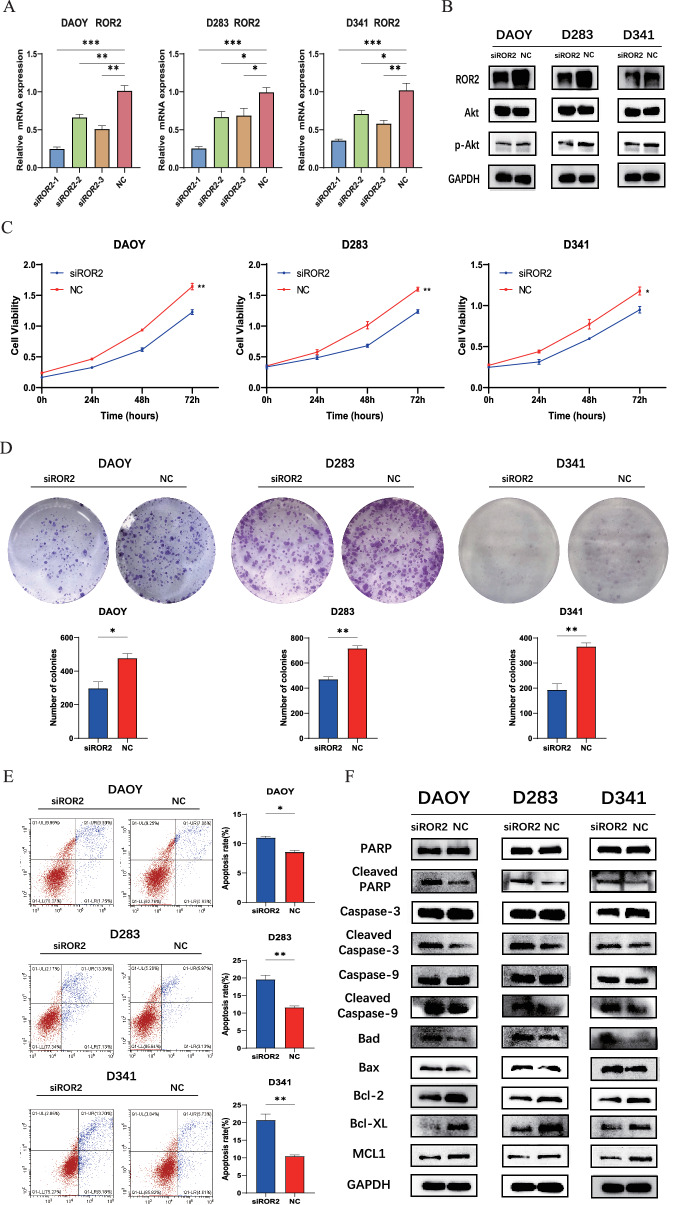
Inhibition of ROR2 suppresses proliferation and induces apoptosis via the PI3K/AKT pathway in MB cells. **A** qRT-PCR analysis of relative ROR2 mRNA expression in MB cell lines transfected with siRNAs. **B** Western blot analysis of ROR2, Akt and p-Akt proteins in MB cell lines. **C**, **D** CCK-8 and colony formation analysis of cell proliferation ability in MB cell lines transfected with siROR2 or NC. **E** Apoptosis analysis of early and late apoptosis in MB cell lines transfected with siROR2 or NC. **F** Western blot analysis of standard apoptosis-related proteins in MB cell lines transfected with siROR2 or NC. Data are shown as mean ± SEM; *P < 0.05, **P < 0.01, ***P < 0.001.

To investigate the potential tumor-promoting role of ROR2 via the PI3K/AKT pathway in MB cells, we designed three specific siRNAs targeting ROR2. Based on the qRT-PCR results, siROR2-1 was selected for subsequent experiments due to high inhibitory efficiency across all three MB cell lines (Fig. 2A). Then, the transfection of siROR2 effectively suppressed ROR2 protein expression in MB cells (Fig. 2B and Supplementary Fig. S1A). ROR2 knockdown led to a reduction in phospho-Akt (Ser473) levels without altering pan-Akt expression in MB cells (Fig. 2B and Supplementary Fig. S1A).

Subsequently, CCK-8 assays demonstrated that downregulating ROR2 significantly inhibited the proliferation of MB cell lines (Fig. 2C). Similarly, colony formation assays revealed pronounced suppression of cell cloning capabilities in response to ROR2 suppression (Fig. 2D). Furthermore, the contribution of apoptosis to the antiproliferative effect of ROR2 was explored. Our results showed that ROR2 knockdown promoted apoptosis in all three MB cell lines (Fig. 2E). Moreover, assessment of standard apoptosis-related markers revealed upregulation of cleaved PARP, cleaved caspase-3, cleaved caspase-9, bad, and bax, while anti-apoptotic markers, including bcl-2, bcl-xl, and mcl1 were suppressed (Fig. 2F and Supplementary Fig. S1B). Together, these results demonstrate the involvement of ROR2 in promoting tumorigenesis through its impact on cell proliferation and apoptosis regulation in MB cells.

### ROR2 enhances migration and invasion of MB cells through epithelial–mesenchymal transition (EMT)

**Fig. 3:**
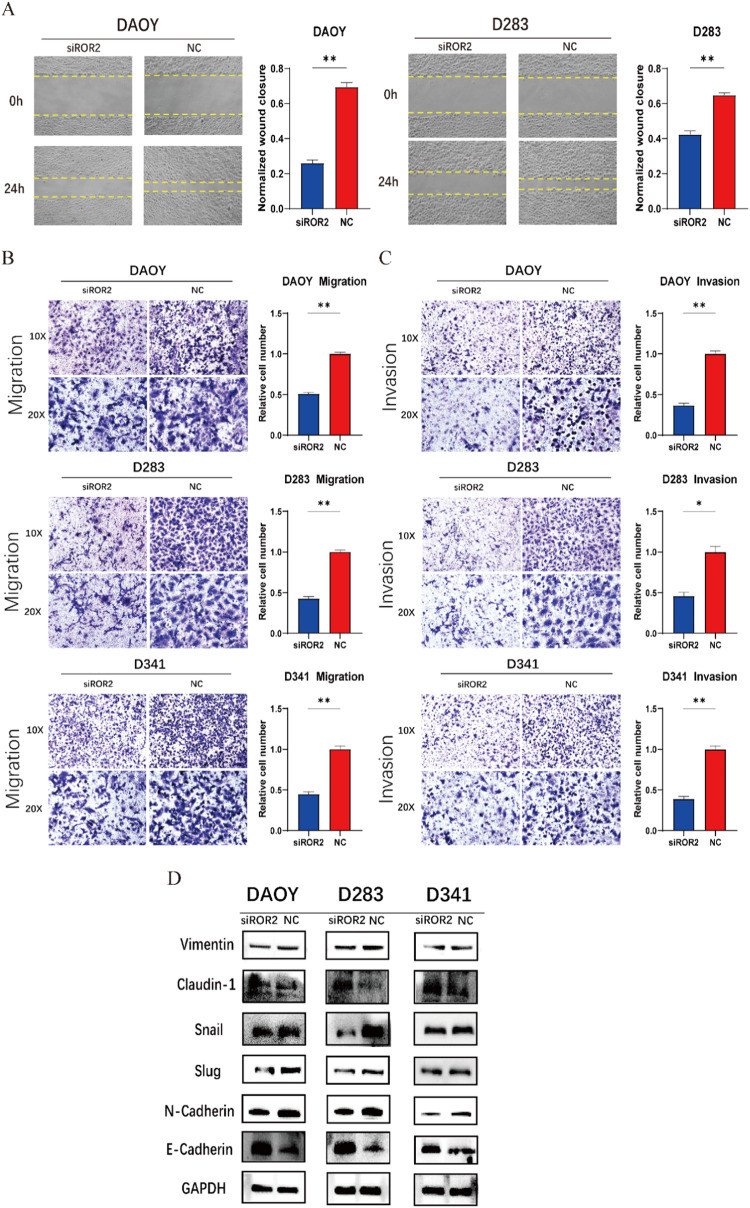
ROR2 enhances migration and invasion of MB cells through epithelial–mesenchymal transition (EMT). **A** Wound healing assay in DAOY and D283 cells transfected with siROR2 or NC. **B**, **C** Transwell migration and invasion assays in MB cell lines transfected with siROR2 or NC. **D** Western blot analysis of standard EMT-related proteins in MB cell lines transfected with siROR2 or NC. Data were showed as mean ± SEM; *P < 0.05, **P < 0.01.

To evaluate the impact of ROR2 on the migratory and invasive capabilities of MB cells, we conducted wound-healing and transwell assays. Silencing ROR2 expression in DAOY and D283 cells resulted in a visibly reduced wound closure area and a corresponding delay in wound closure, indicative of impaired migratory potential (Fig. 3A). Similarly, migration and invasion assays using transwell chambers revealed a significant decrease in the number of tumor cells that traversed to the lower chamber, with or without matrigel, across all three MB cell lines when ROR2 was silenced (Fig. 3B, C).

Given the crucial role of epithelial–mesenchymal transition (EMT) in tumor infiltration, invasion, and metastasis, we examined a panel of EMT-related proteins. WB analysis showed that ROR2 downregulation resulted in decreased expression levels of mesenchymal cell markers and increased expression of epithelial cell markers, including vimentin, claudin-1, snail, slug, N-cadherin, and E-cadherin (Fig. 3D and Supplementary Fig. S1C). Collectively, these findings underscore that ROR2 plays a pivotal role in mediating epithelial–mesenchymal transition (EMT) in MB, influencing tumor invasiveness, metastatic potential, and overall disease progression.

### miR-124-3p and miR-194-5p dually regulate ROR2 in MB cells

**Fig. 4:**
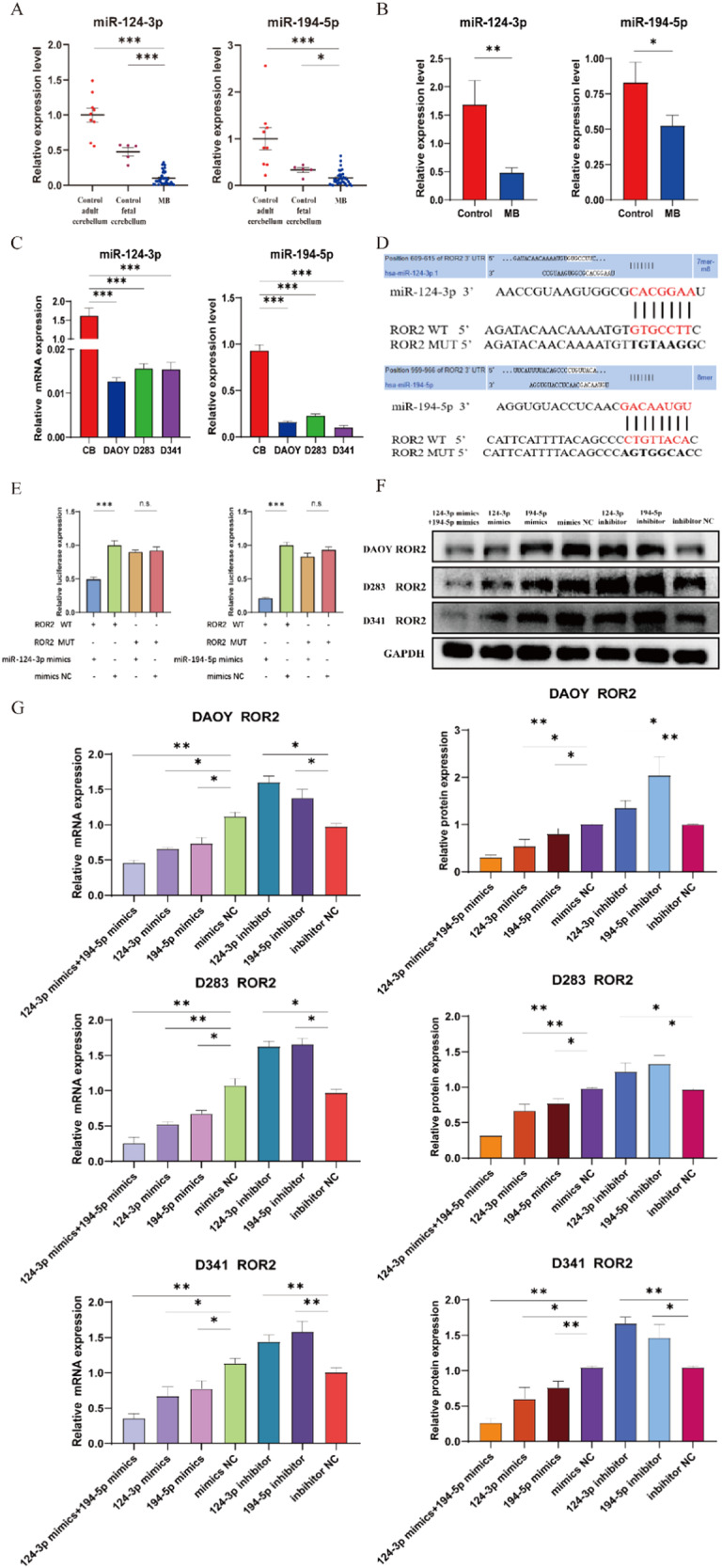
miR-124-3p and miR-194-5p dually regulate ROR2 in MB cells. **A** Expression analysis for miR-124-3p/miR-194-5p in MB and control groups using the GEO2R platform. Ferretti et al., 2021 (*n* = 48, GSE12303). **B** qRT-PCR analysis of relative miR-124-3p/miR-194-5p expression from MB and control tissues. **C** qRT-PCR analysis of relative miR-124-3p/miR-194-5p expression in MB cell lines and normal cerebellum. **D** The potential prediction binding sites between the target gene ROR2 and miR-124-3p/miR-194-5p and the schematic illustration of ROR2-WT/ROR2-MUT dual-luciferase reporter vectors. **E** Dual-luciferase reporter analysis of luciferase activities in cells after co-transfection with ROR2-WT, ROR2-MUT and miR-124-3p/miR-194-5p mimics or mimics NC, respectively. **F**, **G** Western blot and qRT-PCR analysis of ROR2 protein and mRNA in MB cell lines transfected with miR-124-3p mimics, miR-194-5p mimics, mimics NC, miR-124-3p inhibitor, miR-194-5p inhibitor and inhibitor NC, respectively. Data are shown as mean ± SEM; n.s. indicated no significance. *P < 0.05, **P < 0.01, ***P < 0.001.

Given the limited studies investigating the roles of miR-124-3p and miR-194-5p in MB, our investigation was undertaken to unveil their mechanistic and biological implications within MB cells. Initially, through open-source database retrieval, we observed significant downregulation of both miR-124-3p, and miR-194-5p in MB compared to normal cerebellum (Fig. 4A). Analysis of our MB samples corroborated these findings (Fig. 4B). Similarly, at the cellular level, substantial decreases in miR-124-3p and miR-194-5p expression were evident in the DAOY, D283, and D341 cell lines relative to normal cerebellar tissue (Fig. 4C).

To determine if ROR2 is a putative co-regulated target of miR-124-3p and miR-194-5p, bioinformatics tools, including TargetScan, miRanda, and miRmap were employed. Putative binding sites for both miRNAs within ROR2 were identified. Subsequently, we cloned the sequences for ROR2-WT and ROR2-MUT into dual-luciferase reporter vectors, encompassing the predicted binding regions for miR-124-3p and miR-194-5p on ROR2 (Fig. 4D). Then, to elucidate the regulatory relationship, co-transfection experiments were conducted involving the ROR2 vectors along with the miR-124-3p, miR-194-5p, or NC mimics, within MB cells. The experiment results further reinforced this finding, demonstrating a significant reduction in luciferase reporter activity, specifically in the presence of miR-124-3p and miR-194-5p mimics (Fig. 4E). This targeted reduction was abolished when the binding sites on ROR2 were mutated (Fig. 4E), thus establishing the specificity and validity of the miRNA-ROR2 interaction. Through this systematic approach, we confirmed the direct regulatory interaction between miR-124-3p and miR-194-5p and ROR2. The presence of predicted binding sites within the cloned sequences substantiated the putative nature of this interaction.

To elucidate the biological roles of these miRNAs in MB, DAOY, D283, and D341 cells were transfected with miR-124-3p or miR-194-5p mimics and inhibitors. qRT-PCR and WB analyses revealed a significant reduction of ROR2 expression in cells individually transfected with miR-124-3p or miR-194-5p mimics, while noticeable elevation occurred when these miRNAs were inhibited (Fig. 4F, G). Moreover, co-transfection with both miR-124-3p and miR-194-5p mimics synergistically augmented their suppressive impact on ROR2 compared to transfections with individual miRNAs (Fig. 4F, G). Altogether, these results underscore the direct targeting of ROR2 by both miR-124-3p and miR-194-5p, highlighting a dual regulatory mechanism within MB cells.

### Elevated expression of miR-124-3p and miR-194-5p suppresses proliferation and migration in MB cells

**Fig. 5:**
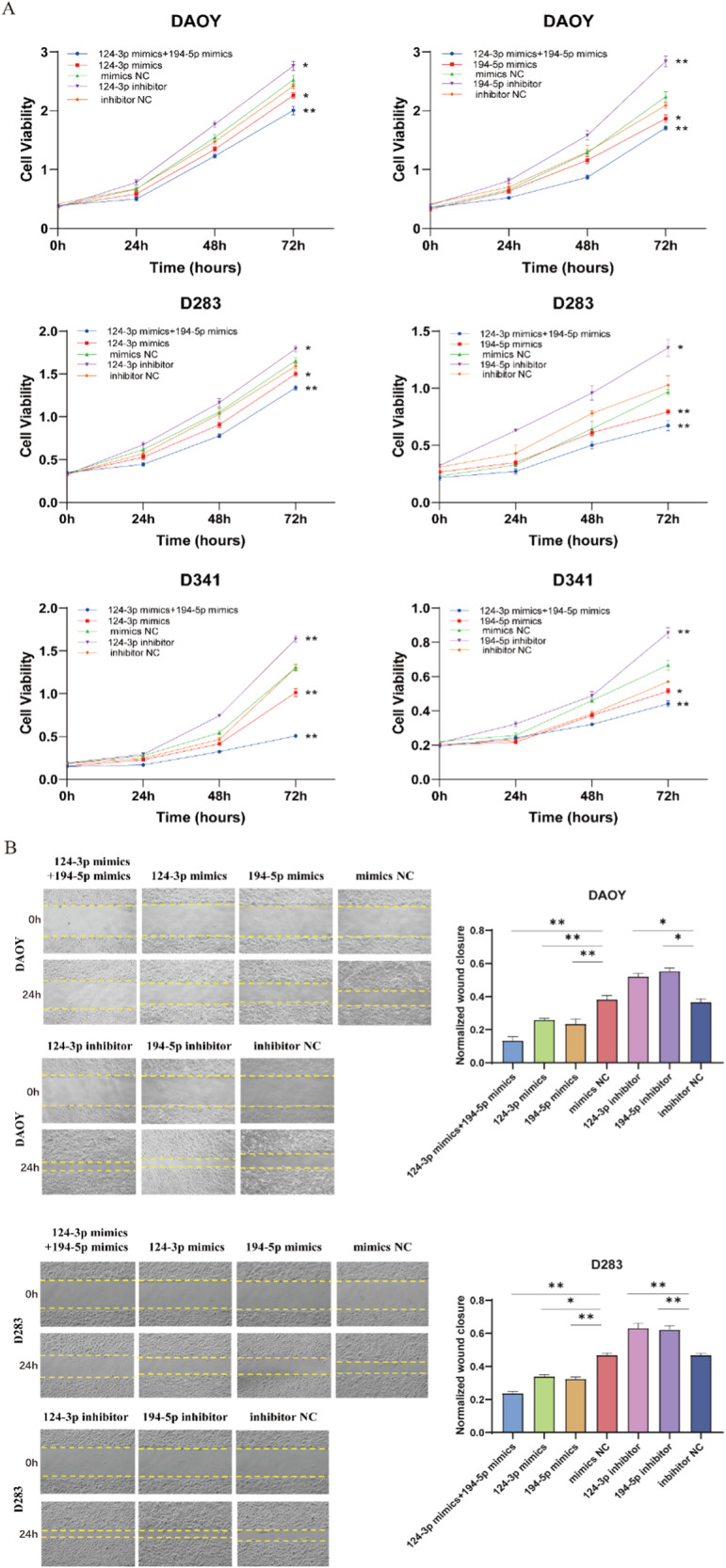
Elevated expression of miR-124-3p and miR-194-5p suppresses proliferation and migration in MB cells. **A** CCK-8 analysis of cell proliferation ability in MB cell lines transfected with miR-124-3p mimics, miR-194-5p mimics, mimics NC, miR-124-3p inhibitor, miR-194-5p inhibitor and inhibitor NC, respectively. **B** Wound-healing assay in DAOY and D283 cells transfected with miR-124-3p mimics, miR-194-5p mimics, mimics NC, miR-124-3p inhibitor, miR-194-5p inhibitor and inhibitor NC, respectively. Data are shown as mean ± SEM; *P < 0.05, **P < 0.01.

To further investigate the tumorigenic role of miR-124-3p and miR-194-5p in MB cells, CCK-8, and wound-healing assays were performed. Elevated expression of both miR-124-3p and miR-194-5p impaired proliferation viability in DAOY, D283, and D341 cells, while decreased expression yielded opposite effects (Fig. 5A). Furthermore, increased individual expression of miR-124-3p and miR-194-5p significantly suppressed proliferative capabilities of DAOY, D283 and D341 cells, with a synergistic effect observed upon co-transfection of both miRNAs (Fig. 5A).

Wound-healing assays were performed to evaluate the influence of miR-124-3p and miR-194-5p on MB cell migration. Results indicated that increased expression of miR-124-3p or miR-194-5p significantly suppressed the migratory abilities of DAOY and D283 cells, whereas decreased expression exhibited the opposite effect (Fig. 5B). The combined transfection of miR-124-3p and miR-194-5p resulted in a greater reduction in migration rates of MB cells compared to transfection with either miRNA alone (Fig. 5B).

### Overexpression of miR-124-3p and miR-194-5p and inhibition of ROR2 suppress the proliferation of MB cells in vivo

**Fig. 6:**
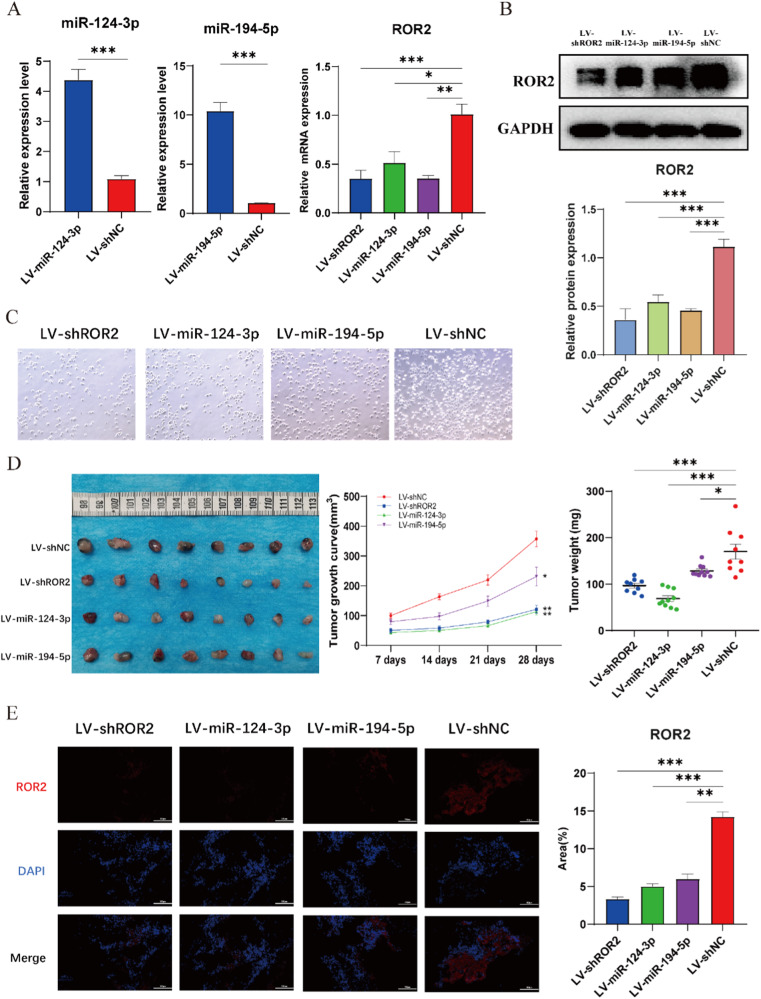
Overexpression of miR-124-3p/miR-194-5p and inhibition of ROR2 suppress the growth of MB in vivo. **A** qRT-PCR analysis of relative miR-124-3p, miR-194-5p and ROR2 expression in DAOY cells stably transfected with miR-124-3p agomir, miR-194-5p agomir, shROR2, and shNC. **B** Western blot analysis of ROR2 protein in DAOY cells stably transfected with miR-124-3p agomir, miR-194-5p agomir, shROR2, and shNC. **C** Proliferation imaging of DAOY cells stably transfected with miR-124-3p agomir, miR-194-5p agomir, shROR2, and shNC after 48 h culture. **D** Image of dissected subcutaneous tumors (left), tumor growth curves and tumor weight analysis (right) from miR-124-3p agomir, miR-194-5p agomir, shROR2 and control group. A ruler was used to indicate the size of the tumors. **E** IF analysis of ROR2 in shROR2, miR-124-3p agomir, miR-194-5p agomir, and control xenograft tumor groups. Data are shown as mean ± SEM; *P < 0.05, **P < 0.01, ***P < 0.001.

To elucidate the in vivo roles of miR-124-3p, miR-194-5p, and ROR2, we established tumor xenograft models using nude mice. DAOY cells were transfected with lentivirus expressing shROR2, miR-124-3p agomir, or miR-194-5p agomir, resulting in the respective inhibition of ROR2 and overexpression of miR-124-3p and miR-194-5p (Fig. 6A), as evidenced by both qRT-PCR and WB assays. Furthermore, decreased expression levels of ROR2 were also observed following transfection with either miRNA (Fig. 6A, B). Cell proliferation evaluation, conducted by imaging cells 48-h post-transfection, revealed impaired proliferative capacities in all three groups of DAOY cells (Fig. 6C), successfully establishing three distinct stably transfected DAOY cell lines.

Subsequently, these cell lines were subcutaneously inoculated into nude mice, with tumor growth monitored for 4 weeks. Tumor volumes were measured every 7 days, and after 28 days, tumor weights were determined. The miR-124-3p and miR-194-5p overexpression groups exhibited significantly reduced tumor volumes and weights compared to the shNC group (Fig. 6D). Similarly, tumors in the shROR2 group displayed a slower growth rate, with significantly lower average volume and weights than those in the shNC group (Fig. 6D). Furthermore, the decreased expression of ROR2 was assessed in xenograft tumors of shROR2, miR-124-3p agomir and miR-194-5p agomir groups by immunofluorescence staining compared to the shNC group (Fig. 6E). Collectively, these findings provide primary evidence that ROR2 functions as a carcinogenic factor promoting MB progression, and its effects are counteracted by the negative regulation of miR-124-3p and miR-194-5p.

## Discussion

MB is a highly aggressive pediatric central nervous system neoplasm characterized by considerable heterogeneity in cell origin, clinical manifestations, disease progression, and treatment outcomes [5]. With recent advances in molecular sequencing, an intricate landscape of DNA copy-number variations, gene transcription profiles, and post-transcriptional modifications within MB has emerged, resulting in an expanded classification of up to 12 molecular subgroups [59]. However, the complexity of these classifications has further complicated the domain of MB therapies.

Our study reveals a pivotal regulatory pathway involving miR-124-3p, miR-194-5p, ROR2, and the PI3K-Akt signaling axis as fundamental drivers of all four core subgroups of MB, holding promising clinical implications for MB management. Utilizing existing drugs targeting ROR2 or components of the PI3K-Akt pathway could expedite translation into clinical trials, capitalizing on their established roles in MB and broader cancer contexts. Moreover, the dysregulation of miR-124-3p and miR-194-5p expression in MB underscores their potential as therapeutic targets, suggesting strategies for their restoration or mimicry. This pathway’s discovery underscores the intricate miRNA-ROR2 interplay in MB growth. Therefore, our study provides essential insights into MB’s molecular underpinnings and illuminates novel therapeutic avenues, potentially shaping more effective strategies against this aggressive pediatric brain tumor.

Central to this paradigm is ROR2, a pleiotropic transmembrane receptor protein with putative kinase activity, which exerts pivotal roles in the pathogenesis of various tumor types [26]. Although ROR2 is dynamically regulated during early embryonic development and exhibits a gradual decline in expression from midgestation onwards, its re-expression or residual presence is associated with aggressive tumor features and poor prognosis in a wide spectrum of malignancies [26, 60, 61]. Some research suggests that the cells-of-origin for the WNT, SHH, and G4 subgroups possibly correspond to radial glia, granule neuron progenitors, and unipolar brush cells, respectively [55, 56]. Furthermore, as embryonic development progresses into the neonatal period, radial glia cells differentiate into astrocytes and oligodendrocytes, contributing to the cellular heterogeneity observed within MB [57, 58].

In our investigation, scRNA-seq analysis of human embryonic cerebellar cell populations revealed characteristic high expression of ROR2 in astrocytes, oligodendrocytes, granule neurons, and unipolar brush cells, which are potential origin cells of the WNT, SHH, and G4 subgroups. The expression of ROR2 was examined across four distinct cohorts encompassing a total of 415 MB samples. Our findings consistently demonstrated significant upregulation of ROR2 in MB tissues relative to control cerebellar samples. Moreover, we extended our analysis to a larger dataset comprising 1286 MB cases spanning the WNT, SHH, G3, and G4 subgroups, further confirming the elevated expression of ROR2 relative to 291 control cases. These outcomes were further validated in our MB samples using IF, qRT-PCR, and WB assays. Moreover, elevated ROR2 levels in cancer patients are associated with unfavorable clinical outcomes, including lower overall survival rates, higher histological grades, worse disease-free survival, and advanced metastatic stages [62–64]. Notably, within the context of the WNT and SHH subgroups of MB, elevated ROR2 expression is significantly correlated with reduced survival durations in affected children. Then, to elucidate the functional roles of ROR2 in MB, a series of targeted experiments were performed.

ROR2 is implicated in carcinogenesis by enhancing various cancer-related features, including cell proliferation, apoptosis, migration, invasion, EMT, and in vivo tumor growth [15]. Given the uncharacterized mechanisms of ROR2 in MB tumorigenesis, we selected three MB cell lines (DAOY, D283, and D341) corresponding to the SHH, G3/4, and G3 subgroups, respectively. The initial verification confirmed elevated expression levels of ROR2 in all three MB cell lines, prompting further investigation into its oncogenic roles. Akt, a phosphoprotein, exerts its cellular effects through phosphorylation-driven conformational changes [65, 66]. Activated p-AKT phosphorylates numerous downstream oncogenic protein effectors [67, 68]. Oncogenic ROR2 overexpression can hyperactivate the PI3K/Akt signaling pathway, contributing to MB progression [69, 70]. Our experiments demonstrated that ROR2 inhibition effectively suppressed p-Akt phosphorylation across all MB cell lines. Subsequently, silencing ROR2 expression promoted apoptosis and inhibited proliferation, migration, and invasion of MB cells in vitro and decelerated MB tumor growth in vivo. Moreover, suppression of ROR2 expression resulted in the upregulation of apoptosis-related and epithelial-associated proteins, concomitant with the downregulation of anti-apoptotic and mesenchymal-linked markers. These combined observations strongly emphasize ROR2’s role as a tumor promotor in all four MB subgroups.

miRNAs have been implicated in oncogenesis for decades, playing a significant role in tumor-suppressive functions [71]. Dysregulated expression of tumor-suppressive miRNAs is closely linked to cancer initiation, progression, and metastasis [72]. Our investigation employed a cohort of 34 MB cases and 14 control cerebellar samples, along with bioinformatics analysis from TargetScan, miRanda, and miRmap databases, to identify potential tumor suppressor miRNAs. We discovered that ROR2 harbored miRNA-binding sites for miR-124-3p and miR-194-5p, both of which exhibited markedly decreased expression in the MB samples. This phenomenon was consistently observed in our MB clinical samples and cell lines, aligning with previous studies [73, 74]. miRNAs exert post-transcriptional regulation by binding to the target mRNA 3’UTR region to suppress gene expression [75]. Dual-luciferase reporter assays were performed, confirming the specific complementary binding and direct regulation between miR-124-3p and miR-194-5p and their target gene ROR2. Recent evidence has highlighted the cancer-related functions of miR-124-3p and miR-194-5p in various central nervous system cancers, such as glioma, ependymoma, and glioblastoma [76–79]. However, despite their significant association with neurons, the roles of miR-124-3p and miR-194-5p as regulators in MB carcinogenesis and progression remain incompletely understood.

Our in vitro results indicated that miR-124-3p and miR-194-5p individually suppressed MB cell proliferation and migration by targeting the ROR2/PI3K/Akt axis. Co-transfection experiments revealed a synergistic enhancement of the inhibitory effects on both growth and migration in MB cells upon simultaneous transfection with miR-124-3p and miR-194-5p, leading to a greater reduction in ROR2 expression. The tumor-suppressive roles of miR-124-3p and miR-194-5p were further validated in our mouse patient-derived xenograft models. In summary, the presented evidence underscores miR-124-3p and miR-194-5p as tumor-suppressive miRNAs, with their decreased expression contributing to MB progression.

In conclusion, our study established a significant link between miR-124-3p and miR-194-5p and their suppressive role in MB growth. Importantly, we revealed that miR-124-3p, miR-194-5p, ROR2, and the PI3K-Akt pathway play a pivotal role in MB progression, underscoring the intricate interplay between these miRNAs and ROR2 expression. Our findings suggest that decreased miR-124-3p and miR-194-5p levels may contribute to ROR2 overexpression, thus promoting MB development. Moving forward, a deeper understanding of the precise molecular mechanisms underlying miR-124-3p and miR-194-5p-mediated regulation of ROR2 is warranted. Although our study focused on the miR-124-3p/miR-194-5p-ROR2-PI3K-Akt axis, it is plausible that other pathways and molecular mechanisms may contribute to MB development and progression. Our insights hold promise for future diagnostic and therapeutic strategies aimed at targeting this regulatory axis to effectively counteract MB progression.

### Supplementary information

Supplementary FigureS1

Supplementary FigureS1 legend

## Data Availability

The datasets used in this study are available from the corresponding author upon reasonable request.
