# Supplementary figures and images for: miR-124-3p and miR-194-5p regulation of the PI3K/AKT pathway via ROR2 in medulloblastoma progression
Source: Cancer Gene Ther. 2024 Mar 19;31(6):941–54. doi: 10.1038/s41417-024-00762-y (PMC11192632; doi:10.1038/s41417-024-00762-y)

**Figure S1**


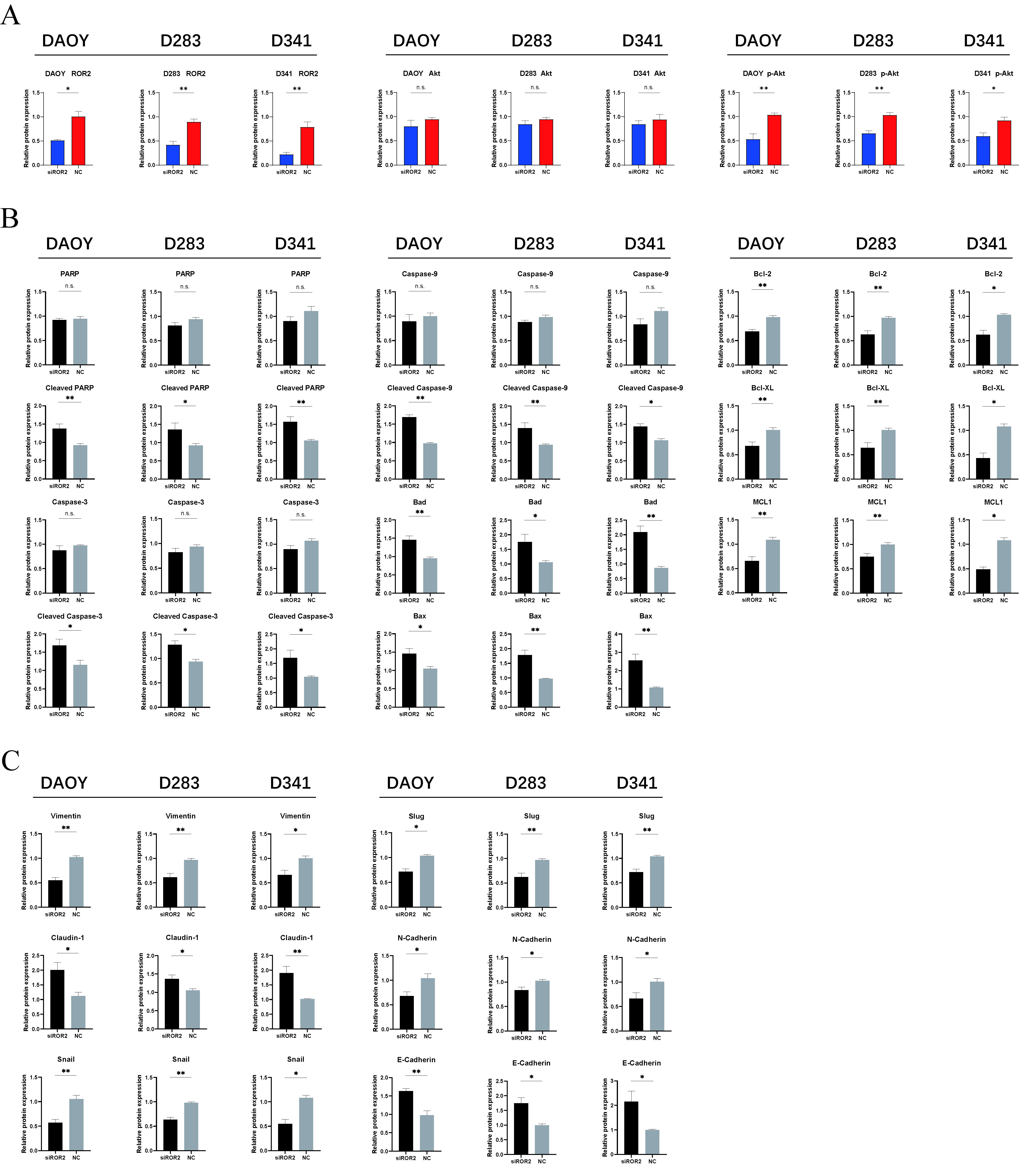

Supplement: Supplementary file 1 — Supplementary FigureS1 [file 41417_2024_762_MOESM1_ESM.docx]
