# Supplementary material for: miR-124-3p and miR-194-5p regulation of the PI3K/AKT pathway via ROR2 in medulloblastoma progression
Source: Cancer Gene Ther. 2024 Mar 19;31(6):941–54. doi: 10.1038/s41417-024-00762-y (PMC11192632; doi:10.1038/s41417-024-00762-y)
Supplement: Supplementary file 2 — Supplementary FigureS1 legend [file 41417_2024_762_MOESM2_ESM.docx]

**Figure S1** Protein levels of Akt, p-Akt, apoptosis-related proteins and EMT-related proteins of MB cells. (**A)** Relative protein levels of ROR2, Akt and p-Akt proteins in MB cell lines transfected with siROR2 or NC. (**B)** Relative protein levels of standard apoptosis-related proteins in MB cell lines transfected with siROR2 or NC. (**C)** Relative protein levels of standard EMT-related proteins in MB cell lines transfected with siROR2 or NC. Data were showed as mean ± SEM; n.s. indicated no significance. *P<0.05, **P<0.01, ***P<0.001.
